# Supplementary material for: Development of Glycosylation-Modified DPPA-1 Compounds as Innovative PD-1/PD-L1 Blockers: Design, Synthesis, and Biological Evaluation
Source: Molecules. 2024 Apr 22;29(8):1898. doi: 10.3390/molecules29081898 (PMC11054459; doi:10.3390/molecules29081898)
Supplement: Supplementary file 1 [file molecules-29-01898-s001.zip › molecules-2889001-supplementary.pdf]

MASS SPECTROMETRY REPORT

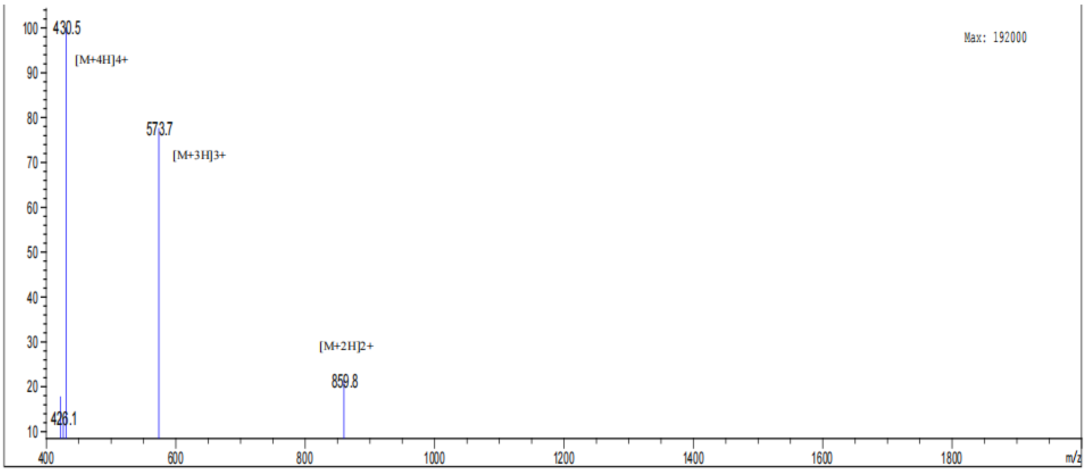

Sample Description		Instrument	Agilent-6125B		
Analyzed date:	2022-02-08	Probe:	ESI	Probe Bias:	+4.5kv
Analyst:	YU	Nebulizer Gas Flow:	1.5L/min	Detector:	1.5kv
Sample:	Glucose-NYSKPTDRQYHF NF-12	CDL:	-20.0v	T. Flow:	0.2ml/min
M.W.:	1717.64	CDL Temp.:	250 °C	B. Conc.:	50%H2O/50%ACN
Lot. No.:	P211213-HS957668	Block Temp.:	200 °C		

MASS SPECTROMETRY REPORT

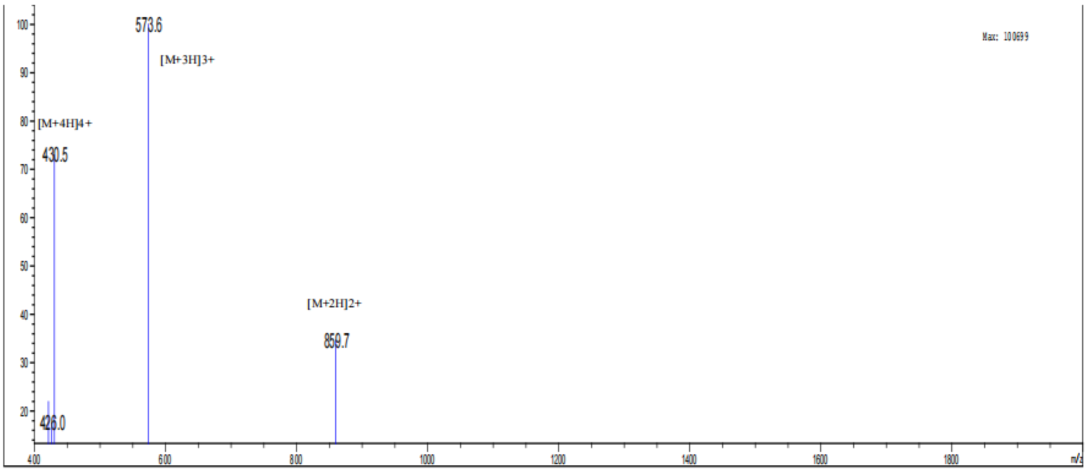

Sample Description		Instrument	Agilent-6125B		
Analyzed date:	2022-02-10	Probe:	ESI	Probe Bias:	+4.5kv
Analyst:	YU	Nebulizer Gas Flow:	1.5L/min	Detector:	1.5kv
Sample:	Gala-NYSKPTDRQYHF NF-12	CDL:	-20.0v	T. Flow:	0.2ml/min
M.W.:	1717.64	CDL Temp.:	250 °C	B. Conc.:	50%H2O/50%ACN
Lot. No.:	P211213-HS957669	Block Temp.:	200 °C		
